# The impact of physicians’ knowledge on outpatient antibiotic use: Evidence from China's county hospitals

**DOI:** 10.1097/MD.0000000000018852

**Published:** 2020-01-17

**Authors:** Haishaerjiang Wushouer, Zhuangfei Wang, Ye Tian, Yue Zhou, Dawei Zhu, Daniel Vuillermin, Luwen Shi, Xiaodong Guan

**Affiliations:** a Center for Strategic Studies, Chinese Academy of Engineering,; b School of Medicine, Tsinghua University,; c International Research Center for Medicinal Administration, Peking University,; d Department of Pharmacy Administration and Clinical Pharmacy, School of Pharmaceutical Sciences, Peking University,; e China Center for Health Development Studies, Peking University,; f Institute for Medical Humanities, Peking University, Beijing, China; g Department of Population Medicine, Harvard Medical School and Harvard Pilgrim Health Care Institute, Boston, MA.

**Keywords:** hospitals, impact, knowledge, physicians, prescribing

## Abstract

We designed this study to explore how factors, especially knowledge, influence the use and prescriptions of antibiotics among physicians in China's county hospitals.

A questionnaire was designed to evaluate the knowledge levels of physicians. The rates of antibiotic prescriptions were collected through on-the-spot investigations. The percentage of encounters with antibiotics prescribed and the percentage of encounters with antibiotics combination prescribed were used to measure antibiotics use. Univariate analysis and the generalized linear model were applied to analyze the knowledge levels among physicians as well as their antibiotic prescriptions.

A total of 334 physicians in 60 county hospitals filled out the questionnaires, and 385,529 prescriptions were collected. The mean score of the questionnaire was a pass (62.8). The physicians in the eastern region of China demonstrated higher levels of knowledge than other regions (*P* = .08). Physicians with a higher score prescribed less antibiotics (*P* < .01) and less antibiotics combination (*P* = .07).

The knowledge gap of Chinese physicians is evident and those with a higher degree of knowledge always prescribe fewer antibiotics. Targeted training and courses to educate physicians about the risks of over-prescription of antibiotics should be conducted to improve the practice of antibiotic prescriptions.

## 1 Introduction

One of the most important factors responsible for the emergence of resistance is irrational use of antibiotics.^[1]^ Study showed that up to one million antibiotics were unnecessarily prescribed each year in the United States,^[2]^ and many antibiotic prescriptions were prescribed for diseases that were not induced by bacteria.^[3]^ The abuse of antibiotics may lead to increased drug expenditure, prolonged hospitalization as well as an increasing social and economic burden on healthcare system and potential antimicrobial resistance (AMR).^[4–7]^

It was estimated that 10 million deaths, of which 4.73 million in Asia, would be attributed to AMR in 2050, and a reduction of 2% to 3.5% in Gross Domestic Product which would cost the world up to 100 trillion USD.^[8]^ In the latest 2 decades, Chinese government endeavored to tackle AMR by adopting different type of interventions, yet overuse and misuse of antibiotics still existed to some extent, especially in the lower level of healthcare institutions.^[9,10]^ Compared with high-income countries, China had a relatively higher rate of antibiotic overuse.^[11]^ The proportion of prescriptions which contain antibiotics was higher in primary healthcare setting than the standard recommended by the World Health Organization (WHO),^[12–14]^ with higher per capita use of antibiotics as well.^[14]^ It turned out that even the prevalence of AMR in China had been moderate in recent years, there is still a long way to go during the journey of tackling the emergence of AMR.^[13]^

Studies identified many factors which would affect the use of antibiotics, such as patient preference or types of insurance.^[15–18]^ However, physicians’ prescription behavior was considered as the primary determinant.^[19]^ Previous research indicated that physicians’ prescribing behavior was influenced by workload,^[20]^ patients’ expectations,^[21]^ medical representatives,^[22]^ and social norm feedback among other factors.^[23]^ Additionally, physicians’ knowledge of antibiotics was an important factor which can be influential on over-prescription. The higher the physicians’ knowledge of antibiotics, the more chances the prescription being rational.^[19,20]^ Thus, there was a significant correlation between physician knowledge and the use of antibiotics.^[19]^

However, literature showed that a large proportion of physicians in lower level healthcare institutions lack academic and professional training in China,^[24]^ which may alter their prescription behavior.^[25]^ As part of the evidence support for decision making in terms of physicians’ knowledge and their prescribing preference in county level hospital in China, we designed this study to explore how factors – in particular knowledge – influence outpatient antibiotic use and prescriptions among county level hospital physicians.

## 2 Methods

### 2.1 Study design

A cross-sectional study was performed to identify the influential factors for outpatient antibiotic use among county level hospital physicians. We designed a questionnaire to evaluate the knowledge levels of the physicians. Outpatient prescriptions of the physicians who completed the questionnaires were collected to analyze the prescribing patterns. Statistical analysis was used to analyze the relationship between the knowledge levels of the physicians and their prescriptions. Ethics committee approval was obtained from Peking University Institution Review Board (IRB00001052-17041) in May 2017.

### 2.2 Questionnaire design

The questionnaire comprised 2 parts. The first part was to collect individual demographic and socioeconomic information, including region, hospital level, department, gender, age, education, professional title, years of working, daily outpatient numbers, personal annual salary and salary satisfaction. The second part was to investigate physicians’ knowledge towards national guidelines and management of antibiotic use as well as clinical knowledge on different types of antibiotics, including 15 single choice questions (single correct answer) and 5 multiple choice questions (multiple correct answers). Single-choice questions were assigned 4 points and multiple-choice questions were assigned 8 points. The full score of the questionnaire was 100. For specific information see in Supplementary file 1.

The questionnaire was designed and optimized by infectious disease physicians and clinical pharmacists from Peking University First Hospital. After conducting three focus groups and a pilot field research study in two hospitals in Beijing, all of the experts agreed that the questionnaire was valid.

### 2.3 Sampling

A stratified cluster randomized sampling method was employed in the study.

#### 2.3.1 Hospitals

Six provinces evenly distributed in the eastern, central, and western regions of China were selected according to China Health Statistics Yearbook.^[26]^ Ten county hospitals in each province were selected to conduct field research.

#### 2.3.2 Departments and physicians

Physicians in pediatric and respiratory departments were selected to fill out the questionnaire considering majority of the antibiotic agents were consumed in these 2 departments.^[27]^ Three physicians from each department were sampled which produced a total of 360 participants.

#### 2.3.3 Prescriptions

If the hospital information system was accessible, all of the prescriptions by participating physicians from 1 June 2015 to 31 May 2016 were collected. If the hospital information system was not accessible, hand-written prescriptions of the physicians who filled out the questionnaires were collected. One prescription of the even days of each month from 1 June 2015 to 31 May 2016 (180 in total) was collected. Prescription information such as the patient's demographic information, prescription date, drug generic name, usage and dosage in conjunction with the name of the prescribing physician was extracted.

### 2.4 Data collecting and quality control

Three trained investigators went to the selected hospitals to conduct the on-site survey and collect the prescriptions from May 2017 to December 2017. All of the questionnaires were completed in 20 minutes without reference to external sources of information and were collected under the supervision of the investigators to ensure accuracy.

The electronic medical records were digitally transferred, while handwritten medical records were collected by taking photos and were manually input to Microsoft Excel and re-checked to minimize errors.

### 2.5 Outcome measure and statistical analysis

According to the WHO International Network for Rational Use of Drugs (INRUD) (World Health Organization., n.d.), we measured antibiotic use by the percentage of prescriptions including antibiotics and percentage of encounters with antibiotic combination prescribed.

Univariate analysis was applied to analyze the knowledge levels of the physicians towards antibiotic use. The determinants of physicians’ antibiotic prescription were analyzed by using the generalized linear model (GLM). The dependent variables were the percentage of encounters with antibiotics prescribed and the percentage of encounters with antibiotics combination prescribed. The independent variables included region, hospital level, department, gender, age, education, professional title, years of working, daily outpatient number, personal annual salary (RMB), and salary satisfaction (Table 1).

**Table 1:**
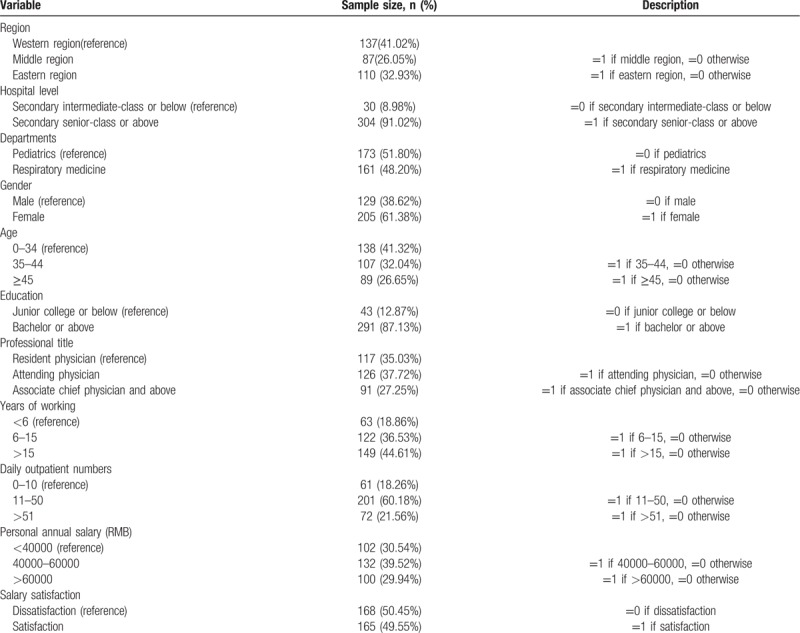
Description and descriptive statistics of physicians (n = 334).

Stata version 14.2 was utilized to clear the original data and perform the data analysis. *P* < .01 was considered statistically significant.

## 3 Results

### 3.1 Demographic information of the physicians

A total of 360 physicians in 60 hospitals were surveyed, of which 334 valid, completed questionnaires were eligible for this study, yielding a response rate of 92.8%. The mean score of the questionnaires was a pass (62.8). 385,529 prescriptions of these 334 physicians were included in the analysis.

These physicians were approximately evenly distributed in different regions across China, and 91.0% of the physicians were from secondary senior-class hospitals or above. 38.6% of the physicians were male. The mean age of the physicians was 39.0. 87.1% of the physicians held an undergraduate degree in medicine. The descriptive statistics of total sample were presented in Table 1.

### 3.2 Antibiotic prescriptions and physicians’ score

**Figure 1:**
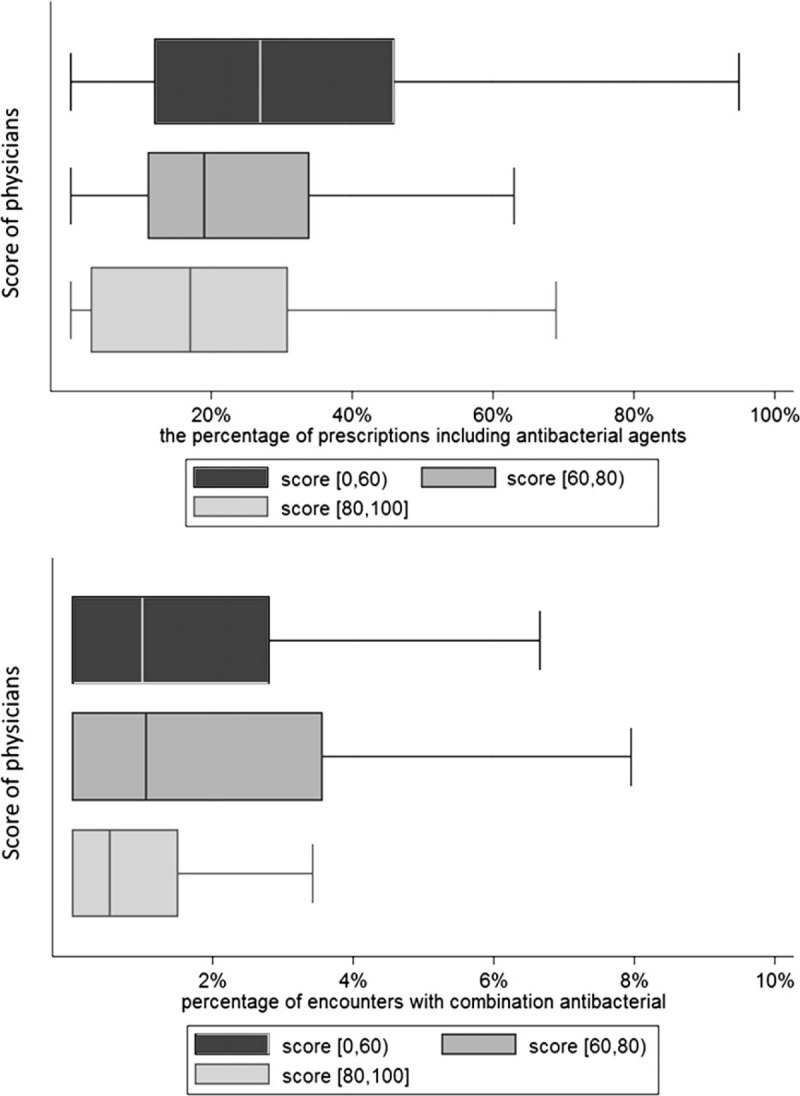
Univariate analysis between antibiotic use and score.

The average percentage of encounters with antibiotics prescribed was 26.8%, while the average percentage of encounters with antibiotics combination prescribed was 2.8%. More details were presented in Supplementary file 2. The physicians were divided into 3 groups according to their score in this section. Physicians who scored in [80–100] group had lower antibiotic prescribing rates than physicians in [60–80) group, and physicians who scored in [0–60) group had highest antibiotic prescribing rates. The percentage of encounters with antibiotics combination in [60–80) group and [0–60) group was relatively close. However, the percentage of encounters with antibiotics combination of the 2 groups was both higher than the physicians in [80–100] group (Fig. 1).

### 3.3 Determinants of physicians’ antibiotic prescriptions

Physicians from the eastern region of China prescribed less antibiotics (*P* < .01) and less antibiotics combination (*P* < .01). Higher level hospitals demonstrated fewer antibiotic prescriptions (*P* < .01). The percentage of encounters with antibiotics prescribed in the respiratory medicine department was lower than those in pediatric departments (*P* < .01), whilst the percentage of encounters with antibiotics combination was higher in respiratory medicine departments (*P* = .08). Larger daily outpatient volume presented higher percentage of encounters with antibiotics (*P* = .05; *P* < .01). Physicians with higher personal annual income prescribed more antibiotics (*P* = .07; *P* = .05) and more antibiotics combination (*P* < .01; *P* = .02). Physicians with a higher score prescribed less antibiotics (*P* < .01) and less antibiotics combination (*P* = .07). Other results of GLM were shown in Table 2.

**Table 2:**
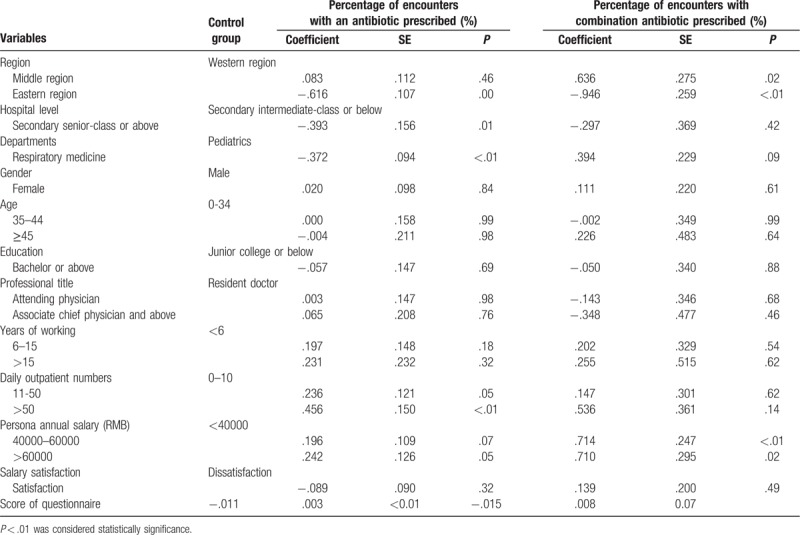
Estimated coefficients of GLM for percentage of encounters with antibiotic prescribed.

## 4 Discussion

Knowledge of antibiotics among physicians is an essential factor affecting the prescription rates of antibiotics. Our study demonstrated that physicians with a higher level of knowledge of antibiotics prescribed less antibiotics and antibiotics combination, which was consistent with the results of studies conducted in other countries.^[28]^

Previous studies showed that Chinese physicians’ prescription behavior may be affected by economic incentives,^[29]^ and physicians were able to make a profit through prescriptions, especially antibiotics, which may have strongly stimulated over-prescription and irrational use of antibiotics.^[30,31]^ However, our study demonstrated that economic incentives did not distort the positive correlation between physicians’ knowledge and antibiotics use. Other than zero mark-up policy implemented towards hospital medication procurement, one possible reason may be that hospitals had strict control over antibiotic use because of the national campaign towards antimicrobial stewardship conducted from 2011 to 2013, which limited the amount of antibiotics that could be prescribed and disciplinary actions caused by the infringement of the antibiotic guidelines would lead to the confinement of the prescribers’ prescription privilege.^[32]^

The low average education of physicians is a remnant of China's complex healthcare system development history. Among all the certificated physicians, nearly half of them did not achieve a bachelor degree.^[33]^ In our study, 13% of physicians’ education background were junior college or below, only 4% of physicians had master degree, and no physicians had doctoral degree. In high-income countries like United States, a doctorate degree is a basic requirement for physicians.^[21]^ Our research found that physicians who had postgraduate degree scored better than those of junior college or below. Many studies demonstrated that continuous education can narrow the knowledge gap among physicians with different levels of education by enriching physicians’ knowledge on antibiotic use,^[34,35]^ which indicated that antibiotic training and courses should be provided on a regular basis. Therefore, training is extremely necessary for Chinese physicians, especially for those with low levels of education.

In addition, training should be targeted. Our research identified distinct knowledge gaps among physicians in different regions. The physicians in the eastern region demonstrated higher level of knowledge (Supplementary file 3), which may attribute to the fact that eastern regions can provide more optimal welfare treatments which in turn might attract more doctors with higher proficiency.^[36]^ Therefore, it is extremely important to enhance physicians’ continuous education in the central and western regions.

We also found that there are other factors that may influence antibiotic use. The first factor is region. As medical resources in China are centralized in the eastern region, western regions are often lack of microbiological testing facilities and expertise of medical staff,^[37]^ which make diagnosis of the physicians from these areas often based on symptoms or just prescribe antibiotics as a prophylaxis. What is worse, sometimes they may try different antibiotics just to cease the symptoms.^[38]^ This might be the reason that physicians in secondary intermediate-class hospitals or below, where medical resources are often limited, would potentially prescribe more antibiotics. Another factor is the hospital department. Antibiotics are among the most commonly prescribed drugs in pediatric department. Physicians are getting used to prescribe antibiotics to treat or prevent infection in a large number of cases which may be caused by non-bacterial pathogens, where the parents of the pediatric patients are more sensible and willing to see immediate recovery.^[39]^ Similarly, antibiotics in respiratory medicine are more common as well due to respiratory diseases are more likely to be infected by bacteria although quite a number of the upper respiratory tract infections are viral.^[40]^ Furthermore, physicians with more daily outpatient volume prescribe more antibiotics. The physicians have an incentive to prescribe antibiotics for it is considered simple and safe to speed up the diagnosis or prevention of complications after many hours of continuous work, which is consistent with decision fatigue.^[41]^

Our study focused on the corresponding relationship between physicians’ knowledge and the prescription rates of antibiotics. The limitation of this study was as follows. First, the data was collected from a cross-sectional survey, indicating that the relationship we found between physicians’ knowledge and antibiotic use was correlative rather than causal. Second, this study adopted 2 indicators - the prescription rate of antibiotics and the prescription rate of antibiotics combination - as proxy for the rationality of physicians’ antibiotic prescriptions, which to certain degree were of representativeness. However, more detailed and patient-centered clinical indicators, such as reference to clinical guidelines was required when assessing rationality of the prescription. In addition, this study included as many factors as possible that may affect the prescription behavior of physicians, yet due to data limitation, some factors were not included in the study, such as the complexity of the diseases, which might cause some bias.

## Acknowledgments

The authors would like to thank the disease experts, physicians and clinical pharmacists from Peking University First Hospital who designed and optimized the questionnaire.

## Author contributions

XG and LS conceptualized and designed the study. HW, ZW and YT screened and completed data extractions. ZW and DZ contributed to analysis of the data. HW, XG, ZW, YT, YZ and DV conducted the final analysis. HW and ZW drafted the initial manuscript. XG and LS drafted subsequent versions. All authors critically reviewed and approved the final version.

Xiaodong GUAN orcid: 0000-0002-1290-3827.
